# Responsiveness to exoskeleton loading during bimanual reaching is associated with corticospinal tract integrity in stroke

**DOI:** 10.3389/fnins.2024.1348103

**Published:** 2024-03-04

**Authors:** Alexander T. Brunfeldt, Barbara S. Bregman, Peter S. Lum

**Affiliations:** ^1^Department of Rehabilitation Medicine, Georgetown University Medical Center, Washington, DC, United States; ^2^MedStar National Rehabilitation Network, Washington, DC, United States; ^3^Department of Biomedical Engineering, The Catholic University of America, Washington, DC, United States

**Keywords:** stroke, upper extremity, neurorehabilitation, electromyography, magnetic resonance imaging

## Abstract

**Background:**

Device-based rehabilitation of upper extremity impairment following stroke often employs one-sized-fits-all approaches that do not account for individual differences in patient characteristics.

**Objective:**

Determine if corticospinal tract lesion load could explain individual differences in the responsiveness to exoskeleton loading of the arms in chronic stroke participants.

**Methods:**

Fourteen stroke participants performed a bimanual shared cursor reaching task in virtual reality while exoskeletons decreased the effective weight of the more-impaired arm and increased the effective weight of the less-impaired arm. We calculated the change in relative displacement between the arms (RC) and the change in relative muscle activity (MC) between the arms from the biceps and deltoids. We calculated corticospinal tract lesion load (wCSTLL) in a subset of 10 participants.

**Results:**

Exoskeleton loading did not change RC (*p* = 0.07) or MC (*p* = 0.47) at the group level, but significant individual differences emerged. Participants with little overlap between the lesion and corticospinal tract responded to loading by decreasing muscle activity in the more-impaired arm relative to the less-impaired arm. The change in deltoid MC was associated with smaller wCSTLL (*R*^2^ = 0.43, *p* = 0.039); there was no such relationship for biceps MC (*R*^2^ < 0.001, *p* = 0.98).

**Conclusion:**

Here we provide evidence that corticospinal tract integrity is a critical feature that determines one’s ability to respond to upper extremity exoskeleton loading. Our work contributes to the development of personalized device-based interventions that would allow clinicians and researchers to titrate constraint levels during bimanual activities.

## 1 Introduction

Stroke is a leading cause of disability worldwide with over 13 million new cases each year (Feigin et al., 2019). Hemiparesis is present in nearly 75% of stroke survivors acutely and between 25 and 50% chronically (Gresham et al., 1996). Upper extremity impairment limits the capacity to perform many everyday activities such as bathing, eating, cleaning, and many recreational activities. Thus, key objectives for stroke survivors are to improve impairments and restore upper extremity function. Currently, the most effective approaches involve rehabilitative therapies. Many traditional therapies focus on restoring strength, flexibility, and control of the more-impaired limb, with little focus on the less-impaired limb (Hatem et al., 2016). For example, constraint-induced movement therapy (CIMT) has patients don a removable cast or mitt on their less-impaired arm, thereby forcing them to use their more-impaired arm (Taub et al., 1994, 1998). Forced-used therapies, like CIMT, significantly restrict bilateral activities, allowing patients to only use their less-impaired hand and arm for crude grasp and postural support. Recent work in both animal models and humans shows advantages to bilateral training compared to unilateral training alone (Kerr et al., 2013; Kantak et al., 2017). The next generation of rehabilitative therapies need to include bilateral training as a core feature; however, recent attempts to translate principles of bilateral motor control into effective treatments is lacking (Kelso et al., 1979; Luft et al., 2004; Whitall et al., 2011). Therefore, a critical next step in designing bilateral therapies for stroke survivors must be to discover the neuromuscular mechanisms subserving bilateral coordination in these patients. Optimal feedback control theory (OFCT) is an attractive framework for uncovering these mechanisms.

A core feature of OFCT is that the motor system tries to optimize motor performance by minimizing movement costs Todorov and Jordan (2002) and Diedrichsen (2007) describes two categories of costs. First, participants minimize the costs associated with the goal of a given task. For example, they try to minimize the distance between the hand and a target object during reaching. And second, participants attempt to “regularize” movements to avoid extraneous or exhausting actions (Diedrichsen et al., 2010; Summerside et al., 2018). While movements outside of the laboratory setting are not always optimal, participants will attempt to minimize these costs, which can lead to a tradeoff between the speed and accuracy of movements (Fitts, 1954). An elegant demonstration of optimization during bimanual reaching uses the shared cursor reaching task, where both arms control a single cursor located at the midpoint between the hands (Diedrichsen, 2007). When a force pushes on one arm, participants use both arms to cooperatively counter the perturbation. This cooperative behavior is considered optimal because it reduces asymmetry between the arms. When stroke patients perform shared-goal tasks, they demonstrate greater interlimb asymmetries and reduced coordination compared to controls, likely reflecting the muscular and control cost discrepancy between the limbs (Lodha et al., 2012; Ranganathan et al., 2019). We sought to determine if reducing the muscular cost discrepancy between the limbs in chronic stroke survivors would result in their increasing the use of their more-impaired arm during bimanual reaching. Therefore, we tested the critical assumption that chronic stroke survivors could optimize reaching during a bimanual shared cursor task.

We had participants reach for targets in a three-dimensional virtual reality (VR) environment with a cursor projected at the midpoint between the hands (Diedrichsen, 2007). We then used custom exoskeletons to apply assistive torque to the more-impaired arm and resistive torque to the less-impaired arm to alter the muscular costs for each arm. We hypothesized that if chronic stroke survivors could optimize reaching during a bimanual shared cursor task, exoskeleton loading would (a) increase the displacement of their more-impaired arm relative to their less-impaired arm, and they would (b) decrease muscle activity in their more-impaired arm relative to their less-impaired arm. These results would confirm that chronic stroke survivors can optimize reaching in response to altered limb dynamics produced by exoskeleton loading. Such outcomes would further suggest that device-based stroke rehabilitation can use the principles of OFCT to drive behavioral change in patients.

Surprisingly, we did not find evidence to support these hypotheses at the group level, but individual differences emerged. Therefore, the aim of our study evolved to investigate individual differences among participants and to determine how these differences may have influenced reaching behavior. Motor deficits in stroke emerge from a complex interaction between the individual, task, and environment (Newell et al., 1989; Newell and Verhoeven, 2017), and recent biomarker studies have identified lesion characteristics that predict upper extremity impairment and functional recovery in stroke (Zhu et al., 2010; Feng et al., 2015; Lin et al., 2019). Lesion load, or the overlap between the lesion and the corticospinal tract (CST) can predict chronic motor impairment measured by the Upper Extremity Fugl-Meyer (UE-FM) (Fugl-Meyer et al., 1975). The UE-FM is a clinical assessment of impairment, with scores ranging from 0 to 66; higher scores indicate less motor impairment. Moreover, lesion load can predict those most likely to recover from therapy (Cassidy et al., 2018). Therefore, we conducted an exploratory analysis using neuroimaging data from a subset of participants from whom we were able to retrospectively acquire clinical diffusion-weighted imaging (DWI). We predicted that participants with small lesion loads would be able to modulate reaching displacement and muscle activity more effectively than those with large lesion loads. We hypothesized that, in response to exoskeleton loading, there would be (a) a positive relationship between the change in relative displacement between the arms and lesion load, and (b) a negative relationship between the change in relative muscle activity between the arms and lesion load. Such results would suggest that CST lesion load may be a predictor of which chronic stroke survivors can optimize reaching resulting from altered limb dynamics and further determine who might respond to device-based rehabilitation.

## 2 Materials and methods

### 2.1 Participants

We recruited a convenience sample of chronic stroke survivors from the Washington, DC Metro Area. Inclusion criteria were (a) a stroke occurring more than 6 months prior to recruitment. (b) The ability to simultaneously raise both hands to eye level and extend both hands to 70% of full arm extension. This criterion was verified by our having the participant practice the VR reaching task described in *2.3 Virtual reality task*; participants could use any shoulder and elbow configuration required to complete the task, and there was no requirement for digit extension. (c) The ability to follow a 2-step command. Exclusion criteria were (a) concurrent injury or neurological condition, other than stroke impairment, limiting upper extremity use or (b) visual hemi-neglect. After obtaining consent from 19 participants, we excluded 5 individuals from all analyses for not meeting the inclusion/exclusion criteria. UE-FM scores were recorded at time of enrollment by a trained clinical research specialist. Stroke type (hemorrhagic vs. ischemic) and location were verified by an independent neurologist on a subset of participants from whom we obtained neuroimaging data (see *2.5 Data analysis – neuroimaging*). All procedures were approved by the MedStar-Georgetown Institutional Review Board.

### 2.2 Experimental setup

Participants were seated in an armless chair 1.5 meters in front of two Oculus Rift VR infrared cameras (Meta Inc., USA). The cameras captured the 3-demensional position of the integrated Oculus Rift headset and Rift Touch controllers. Participants used these controllers to interact with virtual objects projected into a custom virtual reality environment programmed in the Unity video game engine (Unity Technologies, USA). We recorded controller position at 50 Hz. We then attached our custom, bilateral exoskeleton to the upper arms using Velcro straps. Our exoskeleton produces a torque profile that closely matches that of the torque due to gravity on the upper extremities through a range of motion from neutral (anatomic position) to a forward flexion of 120 degrees. Exoskeleton loading decreased the effective weight of the more-impaired arm by 50% and simultaneously increased the effective weight of the less-impaired arm by 50% (see Supplementary material).

Finally, we attached four Trigno wireless electromyography (EMG) sensors (Delsys, Inc. Natick, MA) to the anterior deltoid and short head of the biceps brachii, bilaterally. These sensors measured surface muscle activity at 2000 Hz.

### 2.3 Virtual reality task

**Figure 1:**
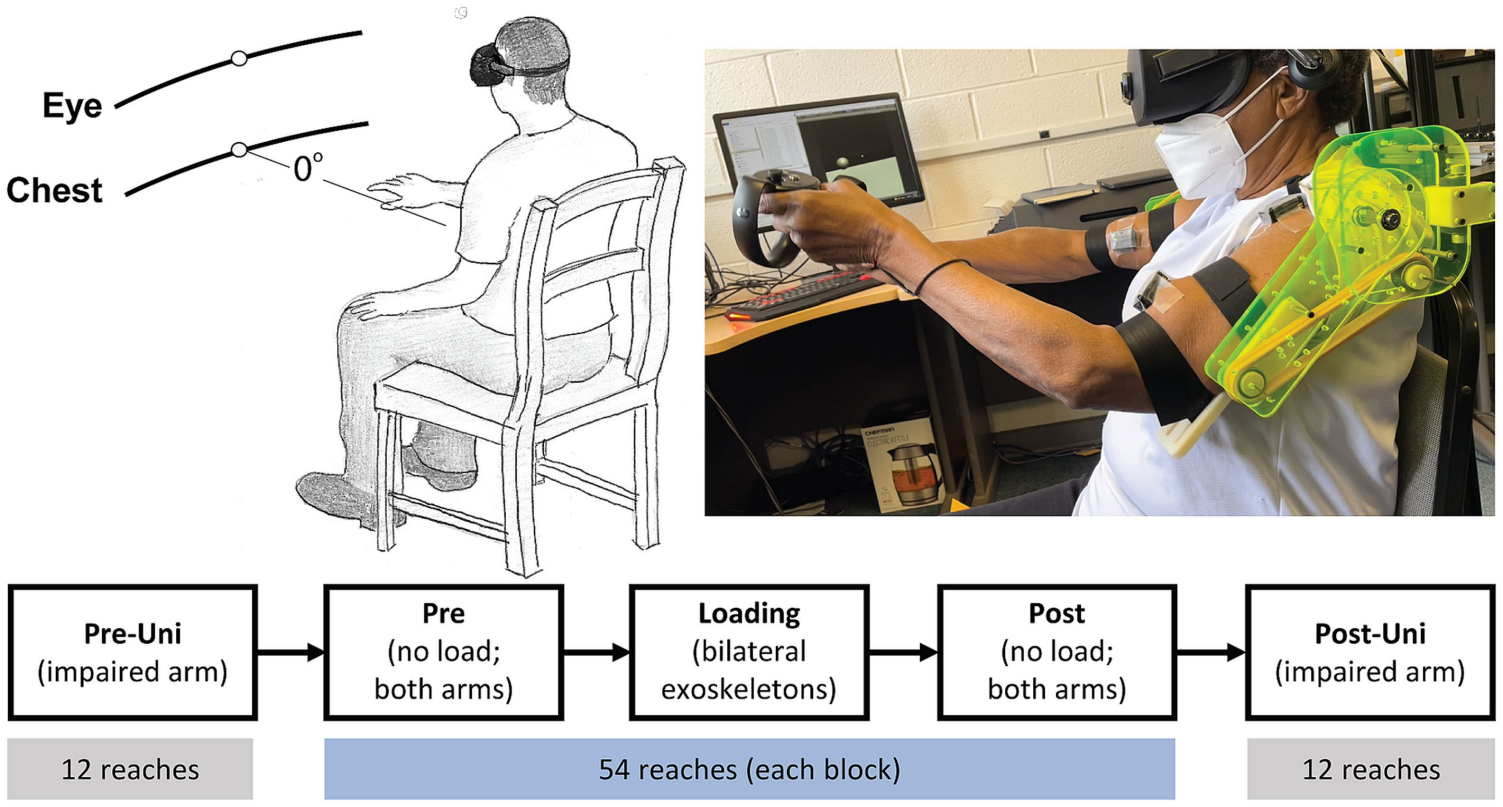
VR target setup (top left), participant reaching with exoskeleton (top right), and task flow (bottom). Pre-Uni and Post-Uni conditions had the participants reach only with their more-impaired arm. Pre, Loading, and Post conditions were bimanual reaches using a shared cursor located at the midpoint between the hands.

Participants reached for virtual cube-shaped targets located at 70% of their maximum arm length. Targets appeared one at a time, at body midline, at either eye or chest level (Figure 1) (Wang et al., 2021; Brunfeldt et al., 2022). Participants controlled a spherical cursor in one of two control modes. In the unimanual mode, the cursor was located at the position of the Rift Touch controller operated by the participant’s more-impaired limb; in the bimanual (shared cursor) mode, the cursor was located at the midpoint between the two controllers. Vision of the hands was not provided. Therefore, the only visual information projected into the VR space was the cursor, target cube, and the default wire grid room. Once a target cube appeared, participants were instructed to move their hand (s) from their lap “as quickly and accurately” as possible to place the cursor inside the cube. Approximately 0.5 s (pseudorandom between 0 and 0.5 s) after the cursor remained inside the target cube, the target cube disappeared, and the participants were instructed to return their hand (s) to their lap. Therefore, one trial comprised the full lap-to-lap reach. Following practice (see Supplementary material), participants performed 5 blocks of trials. The 1st and 5th blocks were unimanual reaches, performed with the more-impaired arm, of 12 trials each; the 2nd and 4th blocks were bimanual reaches without exoskeleton loading; the 3rd block was the bimanual exoskeleton loading condition. Bimanual reaching blocks each had 54 trials. The VR target setup, exoskeleton device, and task flow are illustrated in Figure 1.

### 2.4 Data analysis – VR task

As our primary measure of the kinematic relationship between the arms, we calculated relative contribution (RC) during bimanual blocks as the displacement of the more-impaired hand divided by the sum of displacements of both hands multiplied by 100. The displacement of each hand was defined as the change in 3-dimensional position from trial starting position (hands in lap) to cursor-target collision. An RC value equal to 50% indicates that both arms reach with the same displacement to the target; an RC value less than 50% indicates that the more-impaired arm reaches with less displacement than the less-impaired arm. During unimanual blocks, we calculated the reaction time, movement time, average and peak velocity.

As our primary measure of the dynamic relationship between the arms, we calculated muscle contribution (MC). MC was calculated for each muscle pair (a. deltoid and biceps brachii) by taking the root-mean square (RMS) muscle activity of the more-impaired muscle divided by the sum of RMS values from both muscles. RMS data were conditioned and normalized prior to MC calculation. Specifically, EMG recordings were first detrended and zero-offset, followed by bandpass filtering from 5 to 250 Hz (Nguyen et al., 2017). Then, we calculated the RMS of muscle activity during the full lap-to-lap reach. RMS data from each muscle was normalized to that muscle’s activity during maximum voluntary contraction (MVC_RMS_) (Chang et al., 2013; Chalard et al., 2020). To calculate MVC_RMS_, we instructed each participant to sit at a chair and place their hands under a table such that the elbows were flexed to 90 degrees, palms facing upward. Participants then tried to lift the table (isometric contraction) for 5 s; they repeated this for a total of 3 efforts with 1 min rest between each. After conditioning the EMG timeseries (detrend, zero-offset, filtered) and calculating the RMS of the middle 3 s, we averaged the 3 efforts to obtain MVC_RMS_ for each muscle. RMS muscle activity during the full lap-to-lap reach on each trial was divided by muscle-specific MVC_RMS_. Finally, MC was calculated as the normalized RMS of muscle activity in the more-impaired muscle divided by the sum of normalized RMS muscle activity in both the more- and less-impaired muscle.

Our previous work in shared cursor tasks revealed that the optimal reaching strategy resulted in a tradeoff between kinematic and dynamic control of the arms (Brunfeldt et al., 2022). In the current study, we hypothesized that stroke participants would adopt this same optimal reaching strategy. Therefore, we calculated ΔRC as the change in RC from Pre (block 2) to Loading (block 3); we also calculated ΔMC as the change in MC from Pre to Loading for each muscle pair, respectively. We then created scatter plots displaying ΔRC vs. ΔMC to explore individual differences in motor behavior.

### 2.5 Data analysis – neuroimaging

Four 4 participants (see Table 1) did not have acute MRI scans in the MedStar medical records system and were not included in the neuroimaging analysis. In the remaining 10 participants, we obtained DWI scans acquired as routine standard of care at MedStar Hospitals. In the event of more than one scan existing in the medical record, we chose the scan closest to the participant’s self-reported date of stroke. Scans were downloaded as DICOM format and converted to NIfTI format using MRIcroGL version 1.2.2022 (Neuroimaging Tools & Resources Collaboratory). The first author, ATB, manually traced each participant’s lesion using ITK-SNAP version 3.8; lesion tracing was verified by an independent neurologist. The resulting lesion mask for each participant was then realigned and normalized to the Montreal Neurological Institute (MNI) mni152 standard stereotaxic space and resliced to a 2mm^3^ voxel size using SPM12.

**Table 1:** Demographic characteristics of chronic stroke participants.

Participant	Age (y)	Sex	Dominant hand pre-stroke	More-impaired limb	Time since stroke (y)	UE-FM	Type	Location
1	60	M	R	L	3.5	18	H	BG
2	75	F	R	R	3.5	44	N/A	N/A
3	73	M	R	R	4	43	I	ACA-MCA border
4	40	F	R	R	2	15	H	Parietal
5	57	F	R	R	5	22	I	ICA
6	32	F	R	L	1.5	35	H	BG & Thalamus
7	66	M	L	R	3	59	I	PLIC
8	77	F	R	R	2	36	N/A	N/A
9	55	M	R	R	1.5	55	H	Thalamus
10	74	M	R	L	12	40	H	MCA
11	46	F	R	R	5	24	N/A	N/A
12	46	M	R	R	1.5	39	N/A	N/A
13	56	M	R	R	2	35	I	BG
14	41	M	R	L	1	32	I	PLIC
Mean (SD)	57.0 (14.6)				3.4 (2.8)	35.5 (12.9)		

M, male, F, female; L, left; R, right; H, hemorrhagic; I, ischemic; BG, basal ganglia; ACA, anterior communicating artery; MCA, middle cerebral artery; ICA, internal carotid artery; PLIC, posterior limb of the internal capsule; N/A, not available.

Each participant’s lesion mask was compared to a population-based atlas of the pyramidal tract. The PyT atlas, developed by Chenot et al. (2019) is an atlas derived from 410 healthy participants that contains both the corticospinal and the corticobulbar tracts. We quantified lesion load, the overlap between the lesion and the CST, using weighted CST lesion load (wCSTLL) (Zhu et al., 2010; Feng et al., 2015). wCSTLL is the volumetric (in cm^3^) overlap between the lesion and CST, weighted by the ratio of the cross-sectional area of the CST at the given voxel location (z-slice) of maximum cross-section of the CST. This adjustment controls for the narrowing of the CST at the posterior limb of the internal capsule.

### 2.6 Data reduction and statistical analysis

For unimanual blocks, we performed paired t-tests (Pre-Uni to Post-Uni) on movement time, reaction time, average and peak velocity. For bimanual blocks, we passed the average RC per block into a one-way ANOVA to test within-subjects factor block (Pre, Loading, Post). We also computed a two-way ANOVA on arm displacement with within-subjects factors block and arm, where arm is defined as more-impaired/less-impaired. Separate one-way ANOVAs were used to determine the main effect of block on MC for each muscle pair. We also computed separate two-way ANOVAs on the change in RMS muscle activity with within-subjects factors block and arm. Significant main effects were then assessed using pair-wise comparisons; *p*-values were adjusted using the Tukey method for multiple comparisons. We report effect sizes using generalized effect size (ges) for ANOVAs (Olejnik and Algina, 2003) and Cohen’s *d* for pairwise comparisons.

We determined the relationship between motor performance and lesion characteristics using linear models. We fit individual linear models for response variables ΔRC and ΔMC (from Pre to Loading). Our primary predictor variable was wCSTLL. We also added lesion volume as a predictor variable. Lesion volume was highly skewed (Fischer moment of skewness = 1.17); therefore, we natural log-transformed lesion volume. The resulting distribution had a skewness of less than 0.08. We report model statistics and multiple *R*^2^.

## 3 Results

Demographic information on the 14 stroke participants who completed all aspects of the motor behavior task are presented in Table 1.

### 3.1 Kinematics

**Figure 2:**
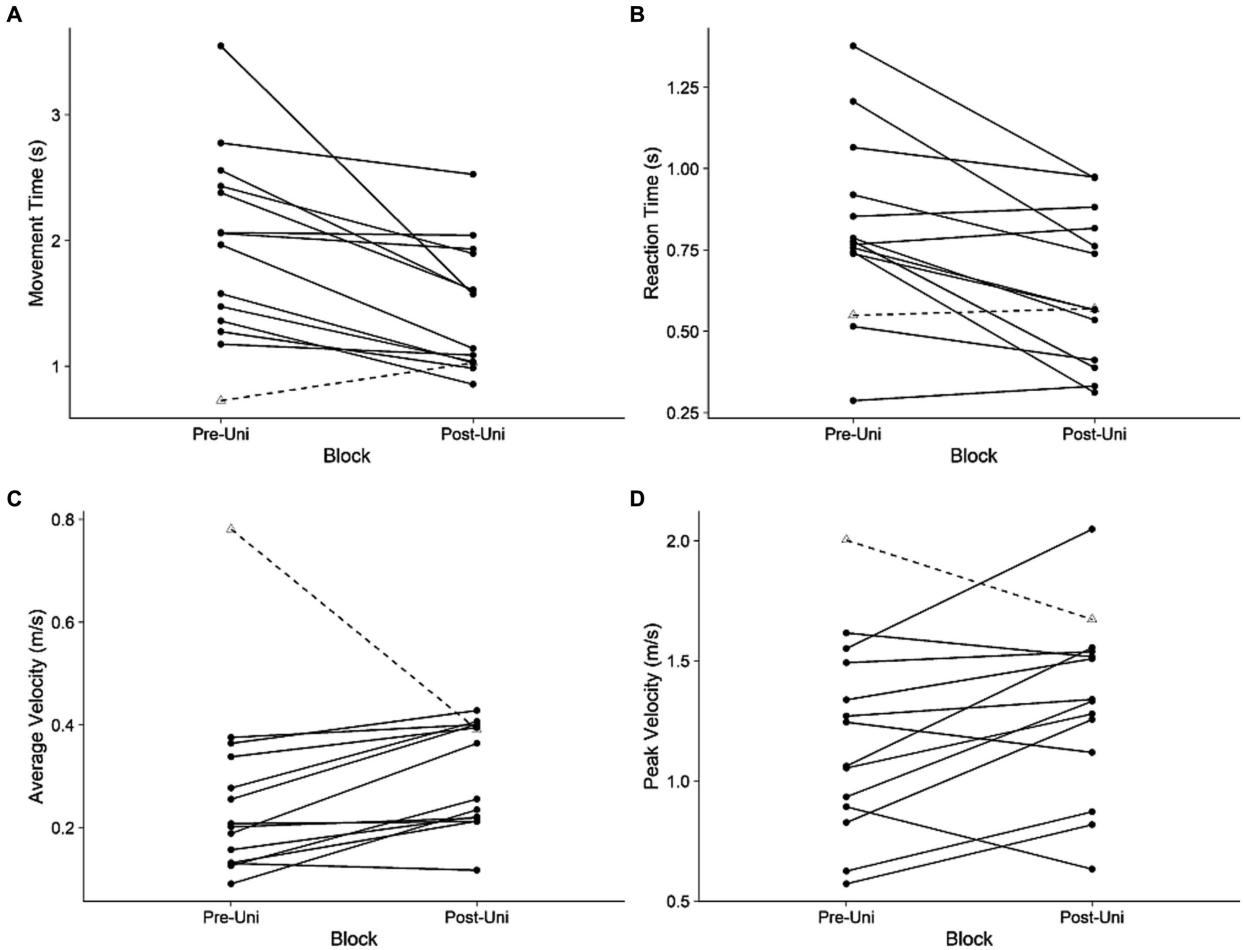
Unimanual kinematics: more-impaired limb. **(A)** Movement time, **(B)** reaction time, **(C)** average velocity, and **(D)** peak velocity during the Pre-Uni and Post-Uni baseline trials. Each line represents an individual participant. Participant 5, who had an unusually large average velocity during the Pre-Uni block, is represented with a dashed line.

Participants moved faster with their more-impaired arm during the Post-Uni block compared to the Pre-Uni block (Figure 2). Both movement time (*t*_13_ = 3.4, *p* = 0.004, *d* = 0.79) and reaction time (*t*_13_ = 3.7, *p* = 0.003, *d* = 0.71) decreased. Peak velocity tended to increase (*t*_13_ = 2.0, *p* = 0.07, *d* = 0.37); average velocity was unchanged (*t*_13_ = 1.2, *p* = 0.24, *d* = 0.31). It is counterintuitive that average velocity did not increase, given that movement time decreased and peak velocity tended to increase. Visual inspection of Figure 2 indicates that one participant (Participant 5) had an unusually large average velocity during the Pre-Uni block. Removing this participant from the analysis yields a significant increase in average velocity for the remaining participants (*t*_12_ = 4.6, *p* = 0.0006, *d* = 0.79).

**Figure 3:**
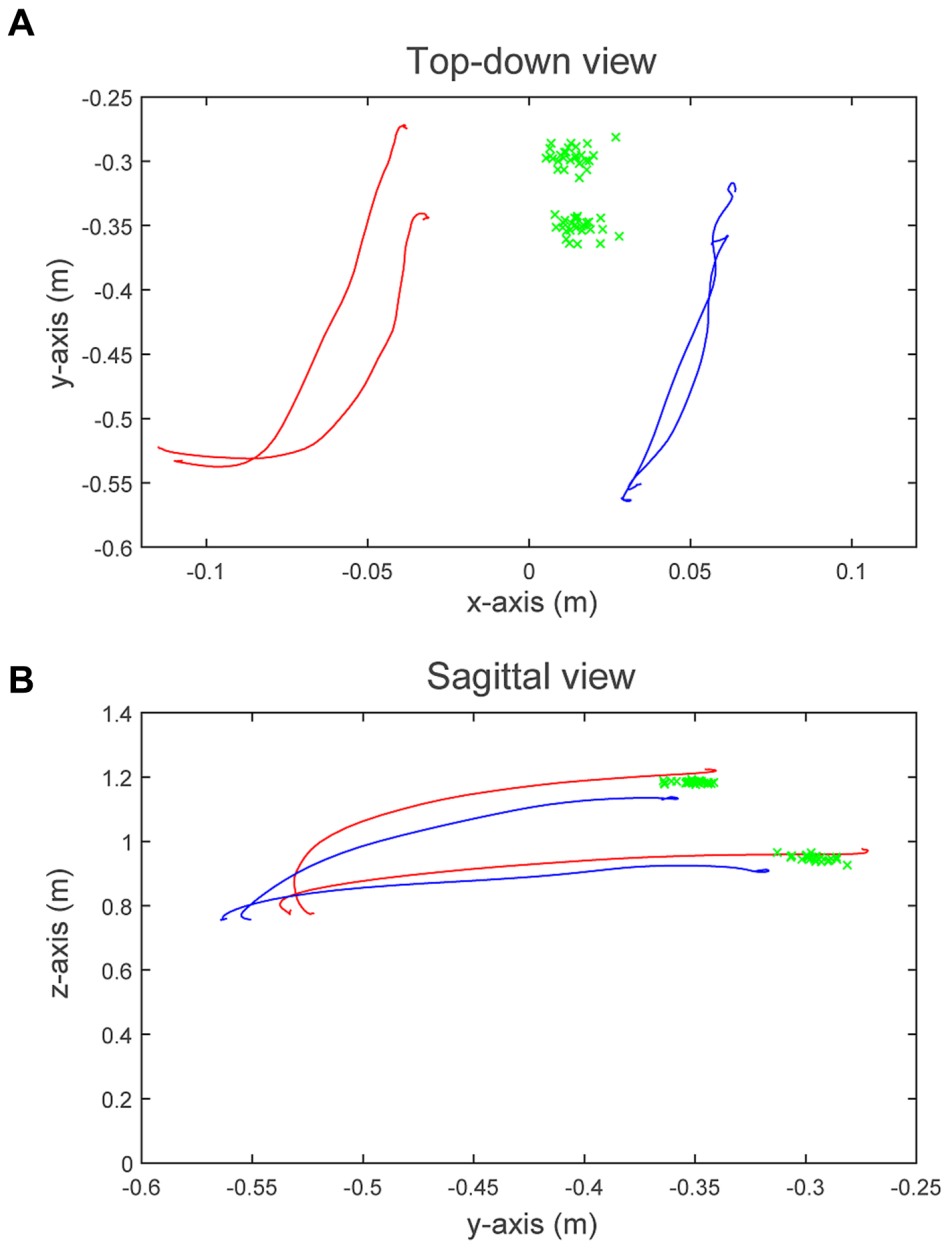
3-Dimensional movement trajectories for Participant 7. **(A)** Displays the transverse plane (from above), and **(B)** displays the sagittal plane (from right-to-left). Blue traces represent the average right hand (more-impaired) trajectories during the Pre loading baseline to the eye- and chest-level targets, respectively; red traces represent the average left hand (less-impaired) trajectories. Green “x”s display the shared-cursor position at target collision for all 54 trials. A plumb line originating at the participant’s head intersects the floor at coordinates (0, −0.75, 0).

Figure 3 illustrates the average 3-dimensional reaching trajectories of one participant (Participant 7) during the Pre loading baseline. To generate these trajectories, we resampled and averaged the 3-dimensional position of each hand, grouped by target location (eye, chest), for all 54 reaches. Figure 3A displays the transverse plane, viewed from above; Figure 3B displays the sagittal plane viewed from the participant’s right-to-left. The participant’s more-impaired right side (blue traces) contributed less to the shared control of the cursor, indicated by the smaller displacement in the right hand (35.0 cm) compared to the left hand (40.9 cm). Average RC was 46.1% for this participant, for this block. For all 14 participants, the average displacement of the more-impaired arm was 8.7 cm in the y-direction (anteriorly) and 26.4 cm in the z-direction (upward from the lap) during the Pre loading baseline. The average displacement of the less-impaired arm was 9.6 cm in the y-direction and 32.7 cm in the z-direction.

**Figure 4:**
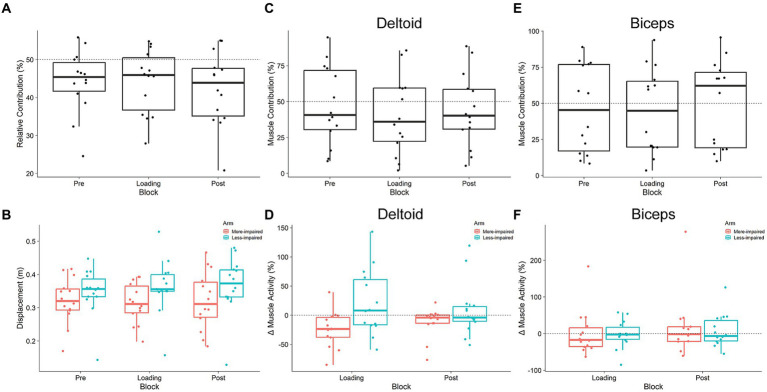
Kinematic **(A,B)** and muscular **(C–F)** relationship between arms during shared cursor reaching. **(A)** Relative Contribution of the more-impaired limb. Values less than 50% indicate stroke participants are reached less with their more-impaired limb. **(B)** Displacement of each arm. Neither arm’s displacement, nor the relationship between them, changed in response to exoskeleton loading (Pre, Loading, Post). **(C,E)** Muscle contribution for the anterior deltoid and biceps brachii in the more-impaired arm relative to the less-impaired arm. **(D,F)** The change (from Pre) in root mean square muscle activity in more-impaired and less-impaired arms for both the anterior deltoid and biceps brachii. Boxplots are in the style of Tukey.

Participants contributed less with their more-impaired arm during the Pre loading baseline (Figure 4A). RC was 44.2%, which was significantly less than 50% (one-sample *t*-test: *t*_13_ = 2.6, *p* = 0.022, *d* = 0.70). There was no main effect of block on RC (F_1.9,24.9_ = 2.97, *p* = 0.07, ges = 0.01). Since RC represents the relative displacement between the limbs, we explored the possibility that the displacement of one arm increased while the other did not (Figure 4B). We did not find a main effect of block on displacement (F_1.61,20.1_ = 1.78, *p* = 0.2, ges = 0.003), nor did we find a main effect of arm (F_1,13_ = 1.52, *p* = 0.24, ges = 0.07). The block x arm interaction was also not significant (F_1.83,23.8_ = 1.88, *p* = 0.18, ges = 0.005). This suggests that, at the group level, our exoskeletons did not significantly change the displacement of either arm or the relative displacement between them.

### 3.2 Muscle activity

Figures 4C,E illustrate the results for MC in both the deltoid and biceps, respectively; Figures 4D,F illustrate the results for RMS muscle activity data. Deltoid MC was no different than 50% during the Pre loading baseline (*t*_13_ = 0.38, *p* = 0.7, *d* = 0.10). Exoskeleton loading did not affect deltoid MC, as there was no main effect of block (F_1.7,22.5_ = 2.16, *p* = 0.144, ges = 0.12). Analysis of the change in RMS deltoid activity from Pre to Loading blocks did not reveal a main effect of block (F_1,13_ = 0.01, *p* = 0.9, ges < 0.001), and there was a trend toward a main effect of arm (more-impaired vs. less-impaired deltoid: F_1.13_ = 3.72, *p* = 0.076, ges = 0.15). There was also a trend toward a block x arm interaction (F_1,13_ = 4.22, *p* = 0.061, ges = 0.24). No post-hoc comparisons on the block x arm interaction trend were significant (all *p* > 0.05). Biceps MC was no different than 50% during the Pre loading baseline (*t*_13_ = 0.48, *p* = 0.64, *d* = 0.12). Exoskeleton loading did not affect biceps MC, as there was no main effect of block (F_1.3,16.3_ = 0.65, *p* = 0.47, ges = 0.35). There was no main effect of block on the change in RMS biceps activity (F_1,13_ = 1.18, *p* = 0.3, ges = 0.02), nor was there a main effect of arm (more-impaired vs. less-impaired biceps: F_1,13_ = 1.1, *p* = 0.313, ges = 0.02). The block x arm interaction was also not significant (F_1,13_ = 1.0, *p* = 0.336, ges = 0.02). Overall, these results suggest that exoskeleton loading did not change motor behavior at the kinematic (RC, displacement) or at the muscular (MC, RMS) level. However, it is common for stroke survivors to display variable behavior, likely due to the heterogeneous nature of their lesion size and location, or the use of compensatory movements (Jayasinghe et al., 2021). Therefore, we used a sensorimotor tradeoff analysis developed by Brunfeldt et al. (2022) to explore individual differences in motor performance.

### 3.3 Tradeoff analysis

**Figure 5:**
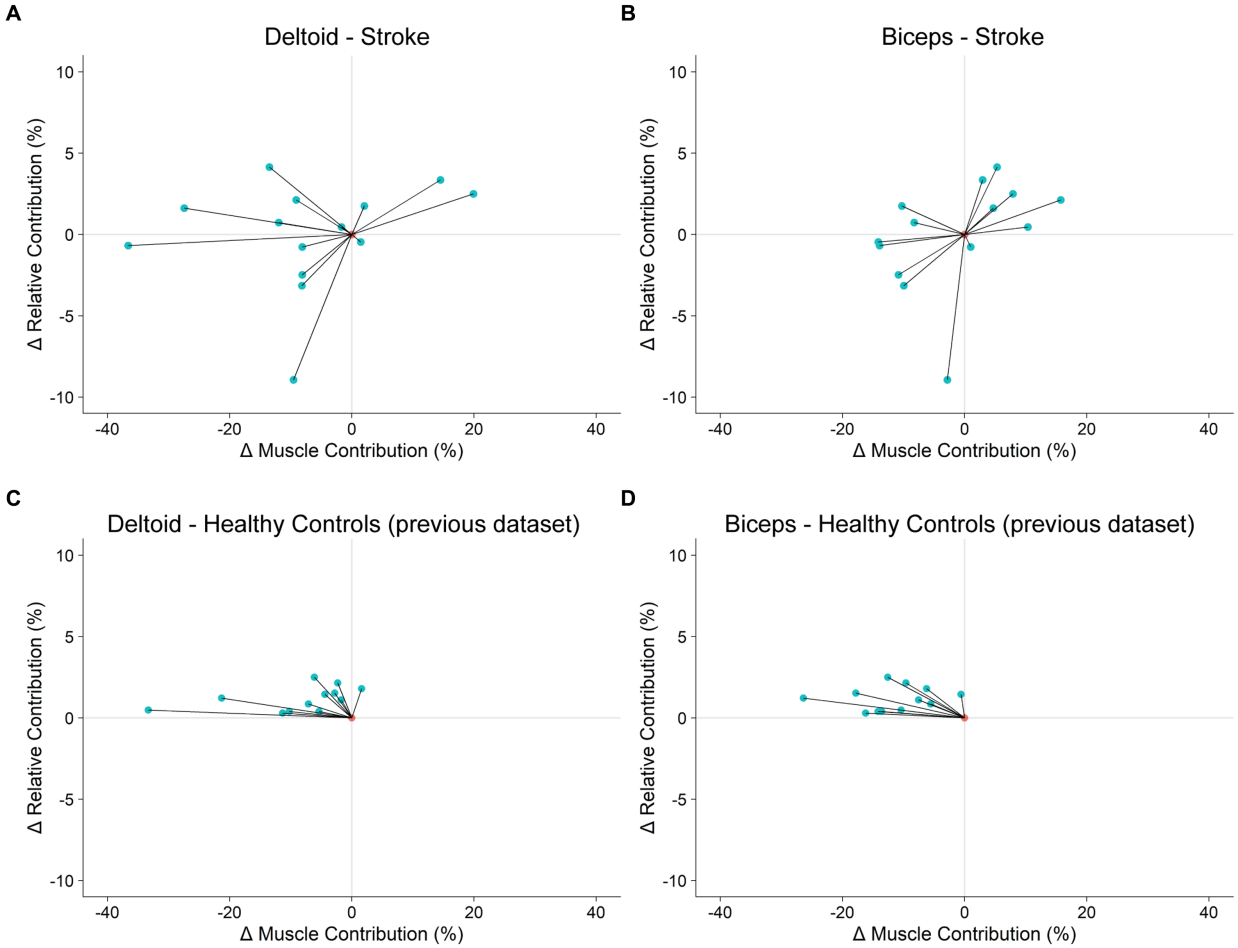
Tradeoff between kinematic and dynamic control of reaching. **(A,B)**: Stroke participants’ change in motor behavior in response to exoskeleton loading. **(C,D)**: Healthy control participants’ change in motor behavior in response to wrist weight loading. Healthy control data were collected in a previous study (Brunfeldt et al., 2022).

We previously found a tradeoff between the kinematic and dynamic control of reaching in healthy individuals (Brunfeldt et al., 2022). Specifically, when we asked healthy young adults to perform our shared cursor reaching task with a wrist weight attached to one arm, RC increased and MC decreased in the non-weighted arm for all 12 participants. Using data from Brunfeldt et al. (2022), we show that when plotting the change in RC (ΔRC) from baseline to wrist weight loading vs. the change in MC (ΔMC), all healthy participants’ behavior occupied the second quadrant (Figures 5C,D). In contrast, our stroke participants display a constellation of responses that occupy nearly all four quadrants (Figures 5A,B). Closer inspection of these data suggests that 5 of our participants adopt an optimal reaching strategy where RC increases and MC decreases in the deltoid of the more-impaired arm (more detail in Supplementary material). Next, we sought to determine if lesion characteristics, specifically the overlap between the lesion and the CST, could account for these individual differences in motor behavior in our sample of chronic stroke survivors.

### 3.4 Lesion characteristics

**Figure 6:**
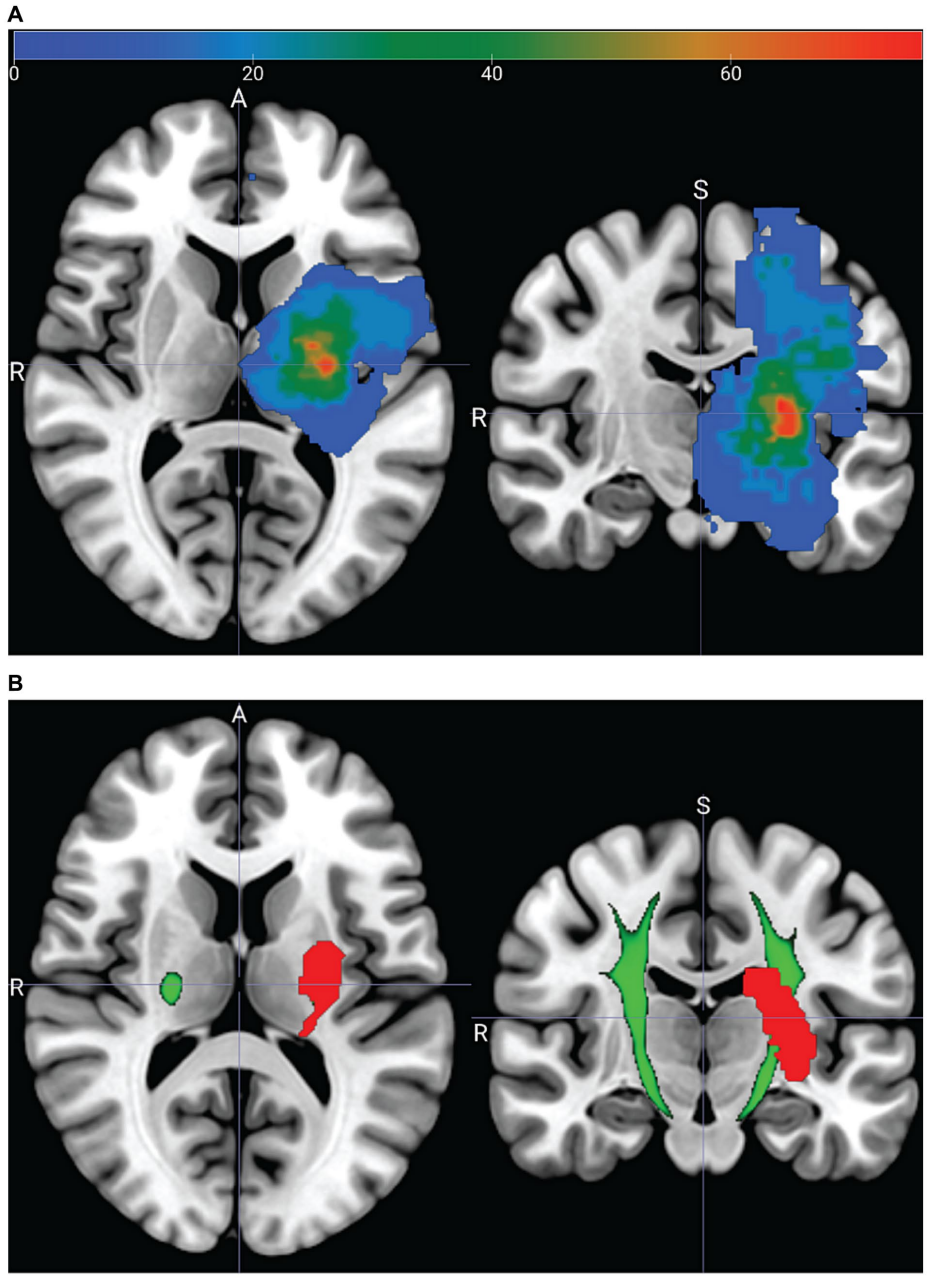
Acute neuroimaging from a subset of 10 stroke participants. **(A)** Lesion heatmap showing the percent (color bar) of participants who had a lesion at a given voxel. Lesion masks from participants with right hemisphere strokes are reflected across the sagittal plane. **(B)** Lesion mask (red), from one participant (13) overlapping the PyT pyramidal tract atlas (green). PyT atlas threshold set to 95%; see Supplementary material – Image processing.

Figure 6A shows a lesion heatmap warped to fit the MNI standard brain template. Visual inspection suggests that the highest lesion density is located near the internal capsule. Figure 6B shows the lesion from one participant overlaid on the PyT pyramidal tract.

**Figure 7:**
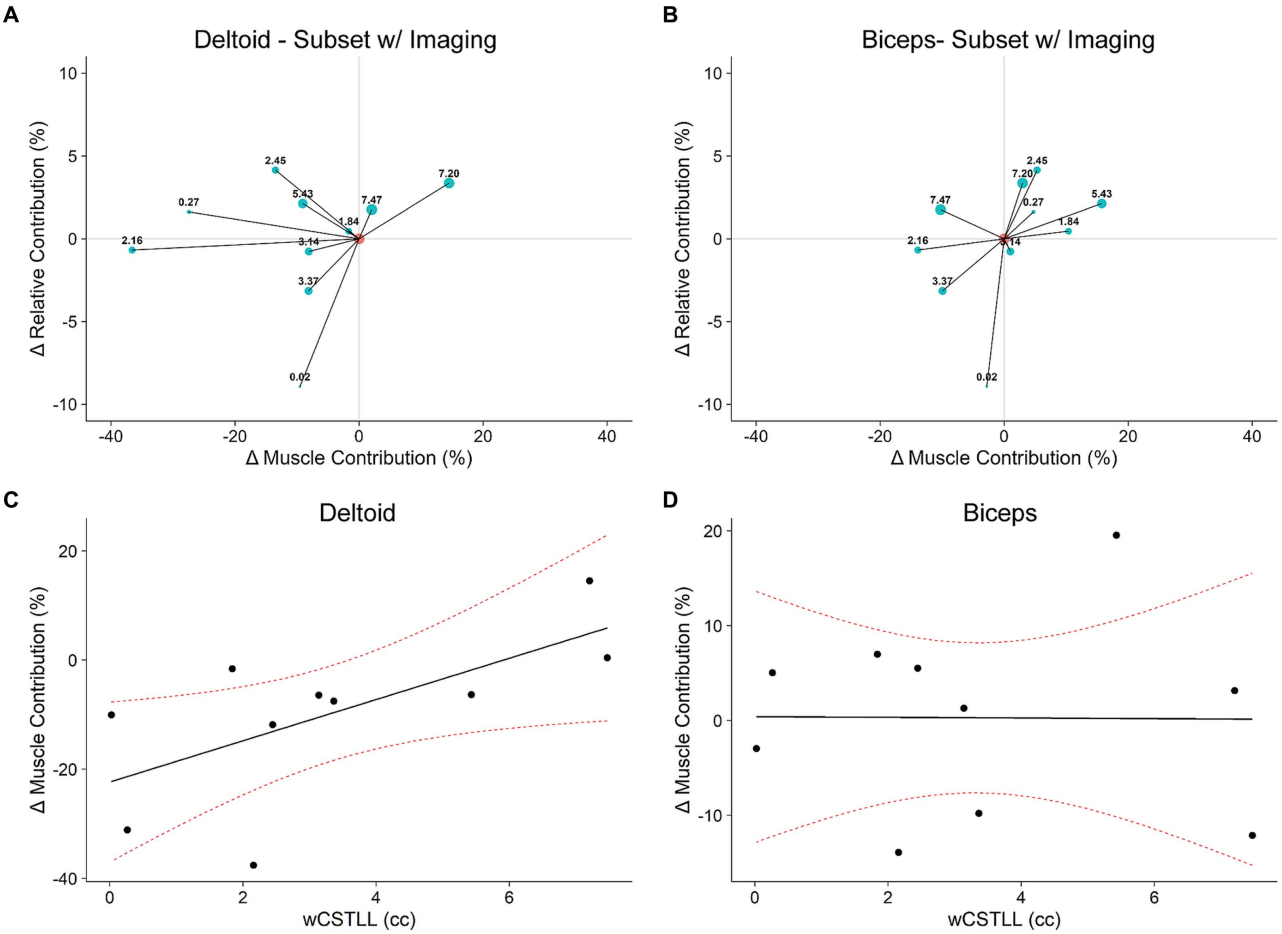
Responsiveness to exoskeleton loading mediated by lesion load. **(A,B)**: Tradeoff plots for a subset of 10 participants with neuroimaging data (see Table 1). Marker sizes and labels corresponds to lesion load (wCSTLL), displayed in cubic centimeters. **(C,D)**: The change in deltoid muscle contribution was larger in participants with more intact CST; this effect is not observed in the biceps. Red dotted curves represent 95% CI.

The relationship between UE-FM and wCSTLL was not significant (*R*^2^ = 0.07, F_1,8_ = 1.3, *p* = 0.22). We then performed an exploratory analysis to determine if motor control outcomes are associated with lesion load. Figures 7A,B show the ΔRC vs. ΔMC tradeoff plots for the deltoid and biceps in the 10 participants with imaging data, respectively; marker size corresponds to wCSTLL. Careful inspection of the deltoid tradeoff plot (Figure 7A) indicates that participants with ΔRC-ΔMC points closer to the y-axis origin (ΔMC = 0) have larger wCSTLL values. Also, 8/10 participants decreased MC in response to exoskeleton loading. That is, the smaller the lesion load, the greater the drop in deltoid MC. To verify this, we fit a linear model to explore the relationship between ΔMC and wCSTLL (Figure 7C). We found that ΔMC in the deltoid was associated with wCSTLL (*R*^2^ = 0.43, F_1,8_ = 6.1, *p* = 0.039), and the intercept was significantly less than zero (*t*_8_ = −3.5, *p* = 0.008). ΔMC in the biceps was not associated with wCSTLL (*R*^2^ = 7.7e-5, F_1,8_ = 6.2e-4, *p* = 0.98) (Figure 7D), nor was ΔRC associated with wCSTLL (*R*^2^ = 0.26, F_1,8_ = 2.9, *p* = 0.13). Log-transformed lesion volume was not a significant predictor of ΔMC in the deltoid (*R*^2^ = 0.02, F_1,8_ = 0.18, *p* = 0.68) nor in the biceps (*R*^2^ = 0.06, F_1,8_ = 0.55, *p* = 0.48). There was not a significant relationship between ΔRC and lesion volume (*R*^2^ = 0.01, F_1,8_ = 0.12, *p* = 0.73). These data suggest that lesion overlap with the CST, and not lesion volume, influences muscle activity during bimanual reaching with exoskeleton devices. Furthermore, the relationship between lesion load and a participant’s muscular response to exoskeleton loading is muscle specific.

## 4 Discussion

In this study, we aimed to determine if chronic stroke participants could optimize motor behavior during a bimanual reaching task. Fourteen chronic stroke survivors reached with both arms to targets in a VR environment while exoskeletons assisted the more-impaired arm and resisted the less-impaired arm. Surprisingly, exoskeleton antigravity support did not change arm kinematics or muscle activity at the group level. Five of the 14 participants increased displacement and decreased muscle activity in the more-impaired arm relative to the less-impaired arm. Instead of a unified response to exoskeleton loading, our findings suggest some stroke survivors adopt alternative strategies. Consequently, our aims evolved to understand why some participants responded to exoskeleton loading while others did not.

We initially expected participants to adopt a strategy consistent with the OFCT (Todorov and Jordan, 2002). According to Diedrichsen (2007), during shared cursor reaching tasks, participants respond to a disruptive force applied to one arm by countering its effects with both arms. The specific response minimizes movement costs because both arms can cooperatively respond to the perturbation. In the current study, we designed our exoskeletons to decrease the movement cost for the more-impaired arm and to increase the movement cost for the less-impaired arm. Fewer than half of the participants optimized reaching via cost minimization, while the remaining participants may have adopted an alternative strategy. Optimal control has been demonstrated in chronic stroke during a shared cursor task (Ranganathan et al., 2019), and in our previous study, all healthy controls optimized reaching by increasing RC and decreasing MC (Brunfeldt et al., 2022). It is possible that participants in our study were not optimizing reaches in terms of reducing effort costs (i.e., energy expenditure), but rather in terms of reducing control costs (Diedrichsen et al., 2010). Gravity support improves unimanual movements in hemiparetic stroke by ameliorating the shoulder-elbow flexor synergy (Twitchell, 1951; Ellis et al., 2005; Beer et al., 2007). Therefore, the ‘optimization’ observed in our study may have reflected a reduction in control costs imposed on shared cursor control by the more-impaired arm. Reducing the flexor synergy may have allowed participants to optimize reaching by facilitating the coordination within- and between arms, rather than through an energy-reduction mechanism. Taken together, the results of our behavioral data do not support the hypothesis that chronic stroke participants optimize shared cursor reaching in response to altered limb dynamics at the group level, but we observed individual differences in interlimb coordination. Therefore, our study aims evolved to determine if lesion characteristics could explain these individual differences.

We observed that the change in deltoid MC was significantly related to wCSTLL. Lesion load has been previously associated with impairment in chronic stroke (Chen et al., 2000; Zhu et al., 2010; Feng et al., 2015; Lin et al., 2019) and the amount of recovery (Riley et al., 2011; Cassidy et al., 2018). Although these biomarker studies imply that damage to the CST disrupts communication between the brain and the limbs, they do not provide a mechanism of action. Participants in our study who had a relatively intact CST were able to reduce deltoid MC in response to exoskeleton loading, but participants with lesions disrupting large portions of the CST did not (Figure 7C). Lesion volume was not related to ΔMC, which echoes the findings in the literature that size is not as important as location when evaluating the relationship between motor outcomes and lesion characteristics (Chen et al., 2000; Heiss and Kidwell, 2014; Feng et al., 2015). We also found that the relationship between ΔMC and wCSTLL was muscle specific (Figures 7C,D). This muscle specific relationship is in accordance with the finding that biceps EMG activity does not correlate with deltoid EMG activity during forward flexion of the shoulder (Gribble and Ostry, 1998). Moreover, the muscle specific relationship is consistent with the altered dynamics of the upper extremity considering the exoskeleton applied a torque about the shoulder, but not the elbow. While we did not explicitly test our participants’ ability to adapt to the novel dynamic environment produced by exoskeleton loading, chronic stroke survivors show a reduced ability to predict the sensory consequences of their movements during force adaptation experiments (Takahashi and Reinkensmeyer, 2003). Therefore, it is possible that CST damage impedes the integration of sensory information into motor action, specifically suggesting that a stroke survivor’s ability to optimize muscle activity in response to altered limb dynamics depends on an intact CST.

Physical and Occupational Therapists have developed rehabilitative therapies focused on behavioral training such as adaptive training, task practice, and operant conditioning over several decades (Taub et al., 1994; Wolf et al., 2006; Schmidt et al., 2018). A recent advancement in upper extremity rehabilitation is CIMT (Taub et al., 1998). These forced-use therapies show superiority over traditional therapy on improving motor function and reducing motor impairment but not on improving disability (Corbetta et al., 2015). CIMT is generally accepted as an effective therapy for cerebral palsy (DeLuca et al., 2012). Barriers to adoption include limited generalizability, resource intensity, and patient-therapist factors (Viana and Teasell, 2012). An opportunity exists in developing patient-focused, customizable constraint-based therapies. The mechanism of action for CIMT centers on movement costs, such as muscular and control efforts. The impairment caused by stroke imposes increased costs on the contralesional limb. Stroke survivors then learn not to use that limb because using the less-impaired limb is less costly (Taub et al., 2006). By placing a cast on the less-impaired limb, movement costs are rebalanced to favor the more-impaired limb. There are two distinct disadvantages to this approach. First, by definition, CIMT restricts many bilateral activities. Recent work suggests that bilateral training may be more effective than unilateral training alone (Kerr et al., 2013; Kantak et al., 2017). Our data reveals that nearly all 14 participants decreased movement and reaction times and increased peak velocity of their more-impaired arm during the Post-Uni block (Figure 2). Shared-cursor tasks could potentially increase movement vigor, thus increasing the likelihood of stroke patients using their more-impaired limb during everyday activities (Summerside et al., 2018; Wang et al., 2021). Second, CIMT is *mechanistically* one-size-fits-all. That is, donning the cast imposes near infinite costs on the less-impaired limb. There is little room to tailor the amount of constraint, beyond the choice of a cast or mitt, based on the individual needs of the patient. Therefore, we propose that bilateral shared-goal tasks in conjunction with customizable device-based interventions paves the way for graded constraint (Huang and Krakauer, 2009; Brunfeldt et al., 2022). Instead of implementing all-or-nothing constraint (Taub et al., 1998), the amount of constraint can be titrated based on individual patient needs. For example, a patient who does not increase displacement or decrease muscle activity during loading may need a higher dose (torque level). The dose could depend on lesion load, and there might be a certain threshold dose that is required to carryover post treatment. Or perhaps the patient’s lesion load is so great that an alternative strategy, such as compensation, is a better choice. Using robotic devices would allow for adaptive constraint, such that constraint is modulated in response to patient performance via principles of progressive overload (Keeling et al., 2021).

Our study has some limitations. First, our data do not include a reliable measure of trunk flexion or shoulder elevation during reaching. The exoskeletons were attached to the testing chair; therefore, trunk and scapular movement was constrained. However, the attachments did allow for some displacement of the shoulder relative to the target cube. Therefore, participants who did not optimize reaching may have adopted an alternative strategy such as motor compensation, or the emergence of alternative coordination patterns resulting from a replacement or substitution of motor elements using different effectors or body parts (Levin et al., 2009). For example, one common motor deficit in stroke is the proximal-distal flexor synergy, whereby stroke survivors unintentionally flex the elbow during forward flexion at the shoulder (Twitchell, 1951). Another deficit in chronic stroke manifests as increased agonist/antagonist co-contraction during reaching (Stoeckmann et al., 2009) which reduces the number of possible muscle combinations available for volitional movements (Dewald et al., 1995). While we did not measure triceps or posterior deltoid EMG, it is possible that our participants had overactive co-contraction which reduced their more-impaired arm’s range of motion. Participants in our study may have compensated for poor upper extremity range of motion by leaning forward with their trunk (Jayasinghe et al., 2021) or elevating the shoulder (Cai et al., 2019) of their more-impaired arm while reaching. Considering the movements were predominately upward from the lap, rather than forward (26–32 cm upward vs. 8–9 cm forward), trunk flexion would be minimal and the shoulder naturally elevates by 5-10 cm during forward flexion of the arm (McClure et al., 2006). Second, there was a large heterogeneity in strokes, with half being ischemic and half hemorrhagic; neuroimaging biomarker literature has been predominantly developed in ischemic stroke (Stinear, 2017). Third, MVC normalization likely underestimates the maximal muscle activation level a participant can exert with their more-impaired arm, which may have resulted in slightly elevated RMS values during reaching in the more-impaired arm compared to the less-impaired arm. This limitation does not affect our interpretation of the MC results, as these measures were baseline corrected (to the “Pre” block), and we employed a within-subjects design. Finally, we could only access neuroimaging data on 10 participants. Although the sample size was limited, we were able to establish a noteworthy connection between ΔMC and wCSTLL. This finding serves as a preliminary indication, supporting the need for further investigation into the influence of CST integrity on how individuals respond to motor adaptation and device-based interventions in stroke. These limitations could be addressed through a prospective cohort study, including chronic ischemic stroke survivors and age-matched controls, with acute DWI neuroimaging as an inclusion criterion.

Our findings have translational potential for rehabilitation. First, our data suggest that an individual’s ability to optimize muscle output in response to exoskeleton loading is associated with CST integrity. Therefore, behavioral assays, used in conjunction with neuroimaging data, may prove a more robust predictor of recovery in stroke. Second, our analyses can be used to assess an individual stroke survivor’s responsiveness to device-based rehabilitation, which may be used by clinical trialists to guide selection and assignment decisions when designing studies. Finally, our tradeoff analysis may be employed to develop graded constraint approaches by determining which stroke survivors can effectively respond to the movement costs associated with goal-directed reaching.

## Data availability statement

The raw data supporting the conclusions of this article will be made available by the authors, without undue reservation.

## Ethics statement

The studies involving humans were approved by Georgetown University Office of Research Oversight/Regulatory Affairs Institutional Review Board. The studies were conducted in accordance with the local legislation and institutional requirements. The participants provided their written informed consent to participate in this study.

## Author contributions

AB: Conceptualization, Data curation, Formal analysis, Funding acquisition, Investigation, Methodology, Project administration, Resources, Software, Supervision, Validation, Visualization, Writing – original draft, Writing – review & editing. BB: Conceptualization, Funding acquisition, Project administration, Resources, Supervision, Writing – review & editing. PL: Conceptualization, Funding acquisition, Project administration, Resources, Supervision, Writing – review & editing.

## References

[ref1] Beer R. F. Ellis M. D. Holubar B. G. Dewald J. P. A. (2007). Impact of gravity loading on post-stroke reaching and its relationship to weakness. Muscle Nerve 36, 242–250. doi: 10.1002/mus.20817, PMID: 17486581 PMC2866301

[ref3] Brunfeldt A. T. Dromerick A. W. Bregman B. S. Lum P. S. (2022). A tradeoff between kinematic and dynamic control of bimanual reaching in virtual reality. J. Neurophysiol. 127, 1279–1288. doi: 10.1152/jn.00461.2021, PMID: 35389759 PMC9054258

[ref4] Cai S. Li G. Zhang X. Huang S. Zheng H. Ma K.. (2019). Detecting compensatory movements of stroke survivors using pressure distribution data and machine learning algorithms. J. Neuroeng. Rehabil. 16:131. doi: 10.1186/s12984-019-0609-6, PMID: 31684970 PMC6829931

[ref5] Cassidy J. M. Tran G. Quinlan E. B. Cramer S. C. (2018). Neuroimaging identifies patients most likely to respond to a restorative stroke therapy. Stroke 49, 433–438. doi: 10.1161/STROKEAHA.117.018844, PMID: 29321336 PMC5780222

[ref6] Chalard A. Belle M. Montané E. Marque P. Amarantini D. Gasq D. (2020). Impact of the EMG normalization method on muscle activation and the antagonist-agonist co-contraction index during active elbow extension: practical implications for post-stroke subjects. J. Electromyogr. Kinesiol. 51:102403. doi: 10.1016/j.jelekin.2020.102403, PMID: 32105912

[ref7] Chang S. H. Durand-Sanchez A. DiTommaso C. Li S. (2013). Interlimb interactions during bilateral voluntary elbow flexion tasks in chronic hemiparetic stroke. Physiol. Rep. 1:e00010. doi: 10.1002/phy2.10 24273652 PMC3831938

[ref8] Chen C. L. Tang F. T. Chen H. C. Chung C. Y. Wong M. K. (2000). Brain lesion size and location: effects on motor recovery and functional outcome in stroke patients. Arch. Phys. Med. Rehabil. 81, 447–452. doi: 10.1053/mr.2000.3837, PMID: 10768534

[ref9] Chenot Q. Tzourio-Mazoyer N. Rheault F. Descoteaux M. Crivello F. Zago L.. (2019). A population-based atlas of the human pyramidal tract in 410 healthy participants. Brain Struct. Funct. 224, 599–612. doi: 10.1007/s00429-018-1798-7, PMID: 30460551

[ref10] Corbetta D. Sirtori V. Castellini G. Moja L. Gatti R. (2015). Constraint-induced movement therapy for upper extremities in people with stroke. Cochrane Database Syst. Rev. 2017:CD004433. doi: 10.1002/14651858.CD004433.pub3, PMID: 26446577 PMC6465192

[ref11] DeLuca S. C. Case-Smith J. Stevenson R. Ramey S. L. (2012). Constraint-induced movement therapy (CIMT) for young children with cerebral palsy: effects of therapeutic dosage. J. Pediatr. Rehabil. Med. 5, 133–142. doi: 10.3233/PRM-2012-0206, PMID: 22699104

[ref12] Dewald J. P. A. Pope P. S. Given J. D. Buchanan T. S. Rymer W. Z. (1995). Abnormal muscle coactivation patterns during isometric torque generation at the elbow and shoulder in hemiparetic subjects. Brain 118, 495–510. doi: 10.1093/brain/118.2.495, PMID: 7735890

[ref13] Diedrichsen J. (2007). Optimal task-dependent changes of bimanual feedback control and adaptation. Curr. Biol. 17, 1675–1679. doi: 10.1016/j.cub.2007.08.051, PMID: 17900901 PMC2230536

[ref14] Diedrichsen J. Shadmehr R. Ivry R. B. (2010). The coordination of movement: optimal feedback control and beyond. Trends Cogn. Sci. 14, 31–39. doi: 10.1016/j.tics.2009.11.004, PMID: 20005767 PMC4350769

[ref15] Ellis M. D. Holubar B. G. Acosta A. M. Beer R. F. Dewald J. P. A. (2005). Modifiability of abnormal isometric elbow and shoulder joint torque coupling after stroke. Muscle Nerve 32, 170–178. doi: 10.1002/mus.20343, PMID: 15880629 PMC2847894

[ref16] Feigin V. L. Nichols E. Alam T. Bannick M. S. Beghi E. Blake N.. (2019). Global, regional, and national burden of neurological disorders, 1990–2016: a systematic analysis for the global burden of disease study 2016. Lancet Neurol. 18, 459–480. doi: 10.1016/S1474-4422(18)30499-X, PMID: 30879893 PMC6459001

[ref17] Feng W. Wang J. Chhatbar P. Y. Doughty C. Landsittel D. Lioutas V. A.. (2015). Corticospinal tract lesion load: an imaging biomarker for stroke motor outcomes: CST lesion load predicts stroke motor outcomes. Ann. Neurol. 78, 860–870. doi: 10.1002/ana.24510, PMID: 26289123 PMC4715758

[ref18] Fitts P. M. (1954). The information capacity of the human motor system in controlling the amplitude of movement. J. Exp. Psychol. 47, 381–391. doi: 10.1037/h0055392, PMID: 13174710

[ref19] Fugl-Meyer A. R. Jaasko L. Leyman I. Olsson S. Steglind S. (1975). The post-Strokeo hemiplegic patient. Scand. J. Rehabil. Med. 7, 13–31. doi: 10.2340/1650197771331, PMID: 1135616

[ref39] Gresham G. E. Duncan P. W. Stason W. B. Adams H. P. Adelman A. M. Alexander D. N. (1996). Post-stroke rehabilitation: assessment, referral, and patient management: quick reference guide for clinicians. J. Pharm. Care Pain Sym. Control. 4, 57–95. doi: 10.1300/J088v04n04_06

[ref20] Gribble P. L. Ostry D. J. (1998). Independent coactivation of shoulder and elbow muscles. Exp. Brain Res. 123, 355–360. doi: 10.1007/s002210050580, PMID: 9860276

[ref21] Hatem S. M. Saussez G. Della Faille M. Prist V. Zhang X. Dispa D.. (2016). Rehabilitation of motor function after stroke: a multiple systematic review focused on techniques to stimulate upper extremity recovery. Front. Hum. Neurosci. 10:442. doi: 10.3389/fnhum.2016.00442 27679565 PMC5020059

[ref22] Heiss W. D. Kidwell C. S. (2014). Imaging for prediction of functional outcome and assessment of recovery in ischemic stroke. Stroke 45, 1195–1201. doi: 10.1161/STROKEAHA.113.003611, PMID: 24595589 PMC3981064

[ref23] Huang V. S. Krakauer J. W. (2009). Robotic neurorehabilitation: a computational motor learning perspective. J. Neuroeng. Rehabil. 6:5. doi: 10.1186/1743-0003-6-5, PMID: 19243614 PMC2653497

[ref24] Jayasinghe S. A. L. Wang R. Gebara R. Biswas S. Ranganathan R. (2021). Compensatory trunk movements in naturalistic reaching and manipulation tasks in chronic stroke survivors. J. Appl. Biomech. 37, 215–223. doi: 10.1123/jab.2020-0090, PMID: 33631718

[ref25] Kantak S. Jax S. Wittenberg G. (2017). Bimanual coordination: a missing piece of arm rehabilitation after stroke. Restor. Neurol. Neurosci. 35, 347–364. doi: 10.3233/RNN-170737, PMID: 28697575

[ref26] Keeling A. B. Piitz M. Semrau J. A. Hill M. D. Scott S. H. Dukelow S. P. (2021). Robot enhanced stroke therapy optimizes rehabilitation (RESTORE): a pilot study. J. Neuroeng. Rehabil. 18:10. doi: 10.1186/s12984-021-00804-8, PMID: 33478563 PMC7819212

[ref27] Kelso J. A. Southard D. L. Goodman D. (1979). On the nature of human interlimb coordination. Science 203, 1029–1031. doi: 10.1126/science.424729 424729

[ref28] Kerr A. L. Wolke M. L. Bell J. A. Jones T. A. (2013). Post-stroke protection from maladaptive effects of learning with the non-paretic forelimb by bimanual home cage experience in C57BL/6 mice. Behav. Brain Res. 252, 180–187. doi: 10.1016/j.bbr.2013.05.062, PMID: 23756140 PMC3742657

[ref30] Levin M. F. Kleim J. A. Wolf S. L. (2009). What do Motor “recovery” and “compensation” mean in patients following stroke? Neurorehabil. Neural Repair 23, 313–319. doi: 10.1177/1545968308328727, PMID: 19118128

[ref31] Lin D. J. Cloutier A. M. Erler K. S. Cassidy J. M. Snider S. B. Ranford J.. (2019). Corticospinal tract injury estimated from acute stroke imaging predicts upper extremity motor recovery after stroke. Stroke 50, 3569–3577. doi: 10.1161/STROKEAHA.119.025898, PMID: 31648631 PMC6878199

[ref32] Lodha N. Coombes S. A. Cauraugh J. H. (2012). Bimanual isometric force control: asymmetry and coordination evidence post stroke. Clin. Neurophysiol. 123, 787–795. doi: 10.1016/j.clinph.2011.08.014, PMID: 21924949

[ref33] Luft A. R. McCombe-Waller S. Whitall J. Forrester L. W. Macko R. Sorkin J. D.. (2004). Repetitive bilateral arm training and motor cortex activation in chronic stroke: a randomized controlled trial. JAMA 292, 1853–1861. doi: 10.1001/jama.292.15.1853, PMID: 15494583 PMC2930817

[ref34] McClure P. W. Michener L. A. Karduna A. R. (2006). Shoulder function and 3-dimensional scapular kinematics in people with and without shoulder impingement syndrome. Phys. Ther. 86, 1075–1090. doi: 10.1093/ptj/86.8.1075 16879042

[ref35] Newell K. M. Van Emmerik R. E. A. McDonald P. V. (1989). Biomechanical constraints and action theory. Hum. Mov. Sci. 8, 403–409. doi: 10.1016/0167-9457(89)90045-6

[ref36] Newell K. M. Verhoeven F. M. (2017). Movement rehabilitation: are the principles of re-learning in the recovery of function the same as those of original learning? Disabil. Rehabil. 39, 121–126. doi: 10.3109/09638288.2016.1170895, PMID: 27268851

[ref37] Nguyen H. B. Lee S. W. Harris-Love M. L. Lum P. S. (2017). Neural coupling between homologous muscles during bimanual tasks: effects of visual and somatosensory feedback. J. Neurophysiol. 117, 655–664. doi: 10.1152/jn.00269.2016, PMID: 27852730 PMC5288480

[ref38] Olejnik S. Algina J. (2003). Generalized eta and omega squared statistics: measures of effect size for some common research designs. Psychol. Methods 8, 434–447. doi: 10.1037/1082-989X.8.4.434, PMID: 14664681

[ref40] Ranganathan R. Gebara R. Andary M. Sylvain J. (2019). Chronic stroke survivors show task-dependent modulation of motor variability during bimanual coordination. J. Neurophysiol. 121, 756–763. doi: 10.1152/jn.00218.2018, PMID: 30601671

[ref41] Riley J. D. Le V. Der-Yeghiaian L. See J. Newton J. M. Ward N. S.. (2011). Anatomy of stroke injury predicts gains from therapy. Stroke 42, 421–426. doi: 10.1161/STROKEAHA.110.599340, PMID: 21164128 PMC3026869

[ref42] Schmidt R. A. Lee T. D. Winstein C. Wulf G. Zelaznik H. N.. (2018) Motor control and learning: a behavioral emphasis. Human Kinetics; Champaign, IL

[ref43] Stinear C. M. (2017). Prediction of motor recovery after stroke: advances in biomarkers. Lancet Neurol. 16, 826–836. doi: 10.1016/S1474-4422(17)30283-1, PMID: 28920888

[ref44] Stoeckmann T. M. Sullivan K. J. Scheidt R. A. (2009). Elastic, viscous, and mass load effects on Poststroke muscle recruitment and co-contraction during reaching: a pilot study. Phys. Ther. 89, 665–678. doi: 10.2522/ptj.20080128, PMID: 19443557 PMC2704029

[ref45] Summerside E. M. Shadmehr R. Ahmed A. A. (2018). Vigor of reaching movements: reward discounts the cost of effort. J. Neurophysiol. 119, 2347–2357. doi: 10.1152/jn.00872.2017, PMID: 29537911 PMC6734091

[ref46] Takahashi C. D. Reinkensmeyer D. J. (2003). Hemiparetic stroke impairs anticipatory control of arm movement. Exp. Brain Res. 149, 131–140. doi: 10.1007/s00221-002-1340-1, PMID: 12610680

[ref47] Taub E. Crago J. E. Burgio L. D. Groomes T. E. Cook E. W. III DeLuca S. C.. (1994). An operant approach to rehabilitation medicine: overcoming learned nonuse by shaping. J. Exp. Anal. Behav. 61, 281–293. doi: 10.1901/jeab.1994.61-281, PMID: 8169577 PMC1334416

[ref48] Taub E. Crago J. E. Uswatte G. (1998). Constraint-induced movement therapy: a new approach to treatment in physical rehabilitation. Rehabil. Psychol. 43, 152–170. doi: 10.1037/0090-5550.43.2.152

[ref49] Taub E. Uswatte G. Mark V. W. Morris D. M. (2006). The learned nonuse phenomenon: implications for rehabilitation. Eura. Medicophys. 42, 241–256. 17039223

[ref50] Todorov E. Jordan M. I. (2002). Optimal feedback control as a theory of motor coordination. Nat. Neurosci. 5, 1226–1235. doi: 10.1038/nn963, PMID: 12404008

[ref51] Twitchell T. E. (1951). THE RESTORATION OF MOTOR FUNCTION FOLLOWING HEMIPLEGIA IN MAN. Brain 74, 443–480. doi: 10.1093/brain/74.4.443, PMID: 14895765

[ref52] Viana R. Teasell R. (2012). Barriers to the implementation of constraint-induced movement therapy into practice. Top. Stroke Rehabil. 19, 104–114. doi: 10.1310/tsr1902-104, PMID: 22436358

[ref53] Wang J. Lum P. S. Shadmehr R. Lee S. W. (2021). Perceived effort affects choice of limb and reaction time of movements. J. Neurophysiol. 125, 63–73. doi: 10.1152/jn.00404.2020, PMID: 33146065 PMC8087386

[ref54] Whitall J. Waller S. M. Sorkin J. D. Forrester L. W. Macko R. F. Hanley D. F.. (2011). Bilateral and unilateral arm training improve motor function through differing Neuroplastic mechanisms: a single-blinded randomized controlled trial. Neurorehabil. Neural Repair 25, 118–129. doi: 10.1177/1545968310380685, PMID: 20930212 PMC3548606

[ref55] Wolf S. L. Winstein C. J. Miller J. P. Taub E. Uswatte G. Morris D.. (2006). Effect of constraint-induced movement therapy on upper extremity function 3 to 9 months after stroke: the EXCITE randomized clinical trial. JAMA 296, 2095–2104. doi: 10.1001/jama.296.17.2095, PMID: 17077374

[ref56] Zhu L. L. Lindenberg R. Alexander M. P. Schlaug G. (2010). Lesion load of the corticospinal tract predicts motor impairment in chronic stroke. Stroke 41, 910–915. doi: 10.1161/STROKEAHA.109.577023, PMID: 20378864 PMC2886713

