# Supplementary material for: Responsiveness to exoskeleton loading during bimanual reaching is associated with corticospinal tract integrity in stroke
Source: Front Neurosci. 2024 Mar 4;18:1348103. doi: 10.3389/fnins.2024.1348103 (PMC10944900; doi:10.3389/fnins.2024.1348103)
Supplement: Supplementary file 1 [file Data_Sheet_1.docx]

Supplement

# Virtual Reality Task

*Participants*

After consenting 19 people, we removed 5 from all analyses for the following reasons: three people were unable to extend the elbow of their more-impaired arm during forward flexion of the shoulder; one participant had a visual hemineglect; one participant could not follow a 2-step command.

*Instructions to participants*

We began the experiment with two practice blocks of 12 trials each. Participants practiced both the unimanual and bimanual reaching modes. In the bimanual mode, we instructed participants to reach first with the left arm, then the right arm, and then both arms. This gave participants explicit knowledge that during the bimanual mode, both arms cooperatively controlled the cursor, and that the most efficient strategy was to use both arms equally. They were verbally instructed to use both arms during the bimanual mode, but that they should “reach however feels most comfortable”.

*Image processing*

Importantly, the PyT tract is probabilistic. That is, each voxel is assigned a value ranging from 0-410 that specifies the number of participants in whom the pyramidal tract is present. Our lesion load metrics (defined below) require a binary tract atlas, so we chose a 95% threshold over which the given voxel was defined as containing the CST (i.e., lesion present in 390/410 atlas participants).

*Statistical analysis*

Visual inspection of MC revealed potential outlier values, likely the result of the EMG sensors interacting with clothing. We removed MC values that fell outside of the median (per block, per muscle) +/- 1.5 IQR, where IQR is the interquartile range (removed 7.4% of observations). We used the same procedure outlined in our prior study (1).

# Exoskeleton Design

We designed our bilateral exoskeleton to apply a torque to the shoulder joint when our participants reached for targets in our virtual reality environment. We used rubber bands to apply force to an upper arm piece that rotated about an axis of rotation pointing through the glenohumeral joint. The mechanics were modelled and tested to ensure the torque profile generated by the exoskeleton matched the torque profile generated by the force due to gravity through a range of motion from neutral (anatomical position) to 120 degrees of forward flexion. Our exoskeletons have two operating modes. In the gravity assistance mode, the torque vector points opposite (180 degrees) that of the gravity torque vector. In the gravity resistance mode, the torque vector points in the same direction (0 degrees) as the gravity torque vector. That is, the torque profiles were equal in magnitude, but opposite in direction. We applied gravity assistance to the more-impaired arm and gravity resistance to the less-impaired arm of our stroke participants. Here we describe the design features, modelling, and testing of the exoskeleton.

*Design and construction*

We designed the exoskeleton in SolidWorks (Dassault Systems, Aachen, Germany). A schematic of the shoulder piece is shown in Figure S1. Planar pieces (e.g., arm piece) were laser cut from 1/4” acrylic. Volumetric pieces (e.g., arm holster) were 3D printed using ABS plastic.

The exoskeleton device was mounted to the participant testing chair using a custom-built frame (see Figure 1 in article text body). The frame was constructed from 1” T-slotted framing rails, which allowed us to raise and lower the exoskeleton device so that its axis of rotation aligned with the glenohumeral joint. We placed the participant’s arm into the holster piece (Figure S1, left panel) and secured it using two Velcro straps. Rubber bands were looped around two needle-roller bearings to reduce friction. These rubber bands produced a force directed along a vector pointing from the distal needle bearing towards the proximal one, indicated by the red dashed line in Figure S1, right panel. In the configuration shown in Figure S1, the rubber bands produced a torque directed against gravity (assist mode). We could move the proximal needle bearing to the inferior portion of the device to produce a torque in the same direction as gravity (resist mode). The number of rubber bands, and the overall mechanical properties of the exoskeleton are modelled and analyzed below.


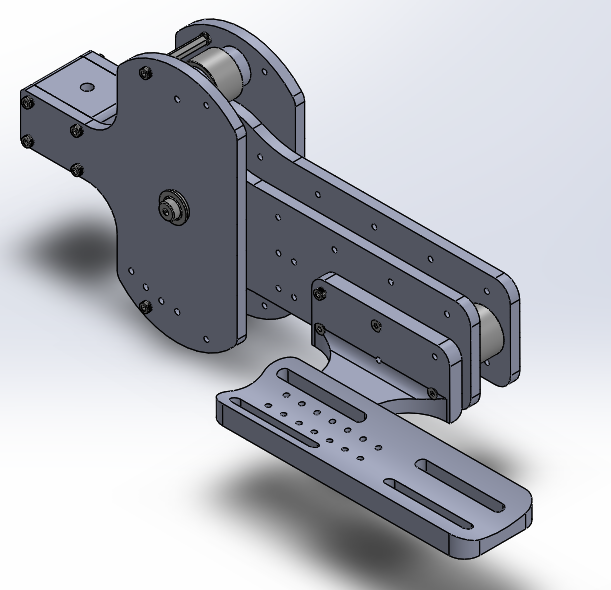

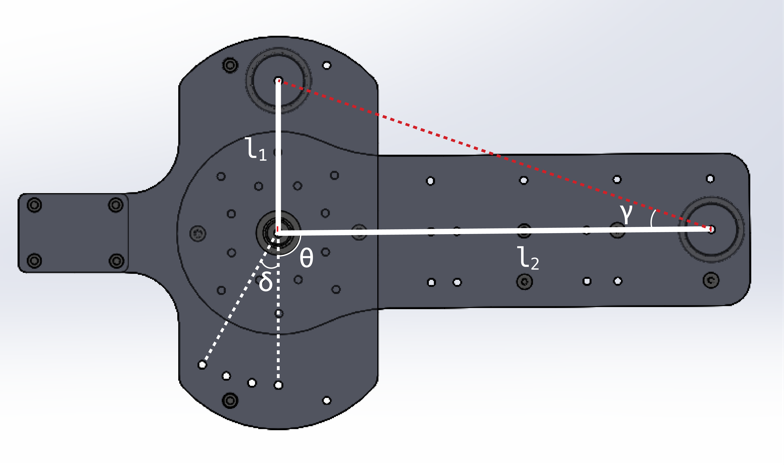


Figure S1: Left shoulder exoskeleton device in assist mode. *Left panel:* Isometric view of the exoskeleton. *Right panel:* Sagittal view of the exoskeleton with arm holster removed.

*Modelling*

We first modeled the torque due to gravity experienced at the shoulder joint during forward flexion of the upper extremity from neutral (anatomical position) to 120 degrees. We assumed the extension occurred with a fully outstretched arm, allowing us to model the torque using a simplified sinusoid function:

*Equation 1*

$$\tau=mgr$$

where g = 9.81 m/s^2^ and r = the moment arm from the glenohumeral joint to the center of mass (COM) of a fully outstretched arm. The constant m; therefore, represents the mass of the upper extremity, located at the COM. For our exemplar modeling illustrated in Figure S2, we selected parameters that would correspond to a 77.1 kg (170 lbs.) participant with an arm length of 0.75 meters. The mass (m) and moment arm of gravitational force (r), would therefore be:

$$m=0.05*77.1 (kg)$$

$$r=0.53*0.75*sin\theta(m)$$

where 0.05 is the percent body weight of the upper extremity and 0.53 defines the location of the COM of the upper extremity expressed as a percent (i.e., 53%) of the distance from the glenohumeral joint to the wrist center. We obtained these from Table 3.2: “Dempster’s Body Segment Parameters” in *Biomechanics of Sport and Exercise* (2). The angle θ (see Figure S1, right panel) represents the arm angle from neutral (anatomical position) through forward flexion. The modeled torque profile is illustrated in Figure S2.

We modeled the mechanical operation of the device in both gravity assistance and resistance modes. The free body diagram of the device yielded the following mechanical properties for the assistance mode:

*Equation 2*

$$\tau=k(x-x_{0})l_{2}sin\gamma$$

where k is some arbitrary spring constant in N/m of the rubber bands; x and x_0_ are the length and rest length, respectively, of the rubber bands; $l_{2}$ is the length of the arm piece (see Figure S1, right panel); $\gamma$ is the angle between the rubber band force vector and the arm piece.

The length of the rubber band, x, changes length as the arm traverses through forward flexion, expressed by the angle θ:

*Equation 3*

$$x= \sqrt{l_{1}+l_{2}-2l_{1}l_{2}\cos\left( 180-\theta\right)}$$

where $l_{1}$ is the length of the shoulder offset (see Figure S1, right panel). The angle defining the moment arm of the rubber band force (angle between rubber band force vector and the arm piece), $\gamma$, was modeled as:

*Equation 4*

$$\gamma= \cos^{-1} \frac{l_{2}^{2}+x^{2}-l_{1}^{2}}{2l_{2}x}$$

For resistance mode, the torque equation (Equation 3) can remain the same, given that the definition of $x$ changes by replacing $\cos\left( 180-\theta\right)$ in Equation 3 with $\cos\left( \theta\right)$. When testing the first designs on pilot participants, we noticed the needle bearings used to secure the rubber bands in place were preventing the participants from fully extending their arm to neutral (0 degrees). We added material in the inferior and posterior aspects of the shoulder piece (see Figure S1, right panel) and machined holes in a radial pattern. Therefore, we could add an angle offset, $\delta$, that would allow for full range of motion. This slightly altered the equation to model the length of the rubber band, x:

*Equation 5*

$$x= \sqrt{l_{1}+l_{2}-2l_{1}l_{2}\cos\left( \theta+\delta\right)}$$

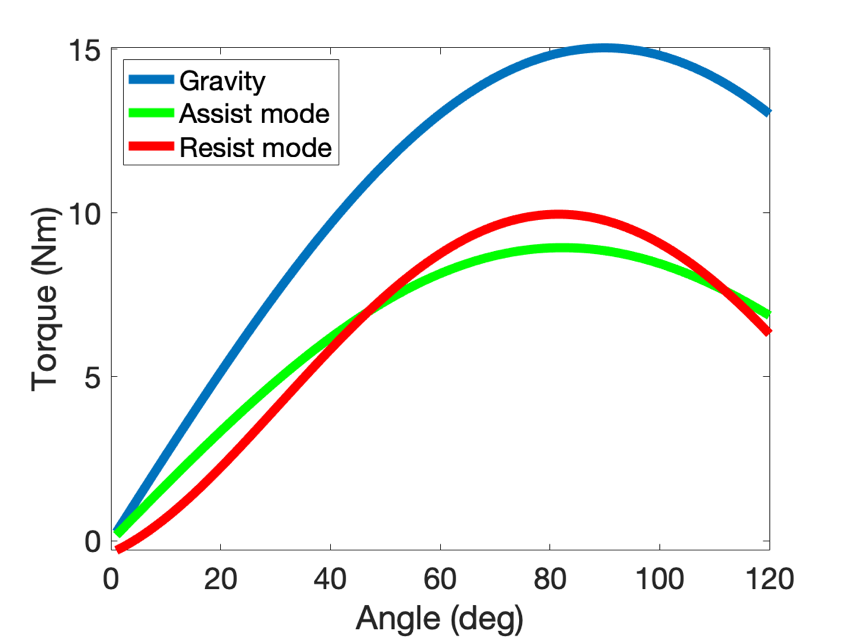


Figure S2: Modeled torque profile from the anatomical position (0 deg) through forward flexion of a fully outstretched arm. Torques are illustrated as the magnitude of torque at a given angle, but the direction depended on torque mode. The direction of torque in assist mode directly countered torque due to gravity; the direction of torque in resist mode was in the same direction as gravity. Both assist and resist mode parameters were plotted to signify an approximately 50% gravity compensation.

*Mechanical testing*

Our goal was to determine the requisite number of rubber bands needed to produce a maximum torque equal to 50% of the torque due to gravity while the arm was held at 90 degrees (horizontal to ground). To calculate these rubber band values, we tested the mechanics of the device by measuring the force (and therefore torque) as a function of flexion angle for several rubber band configurations.

We performed mechanical testing by measuring the force required to move the exoskeleton through a 120 degree range of motion in both assist and resist mode. We measured the force using a 50 lbs. load cell (Transducer Techniques, ML-50) and measured the angle using a potentiometer (Bourns, 3590S-502L 5K Ohm) calibrated to the exoskeleton arm piece angle. Both signals were digitized by a National Instruments Data Acquisition board (NI USB-6009) and analyzed offline using a custom MATLAB script. An experimenter (ATB) moved the arm piece through the full range of motion (0-120 degrees) by manually pulling on the load cell against the torque generated from the rubber bands. Exemplar torque profiles are shown in Figure S3.


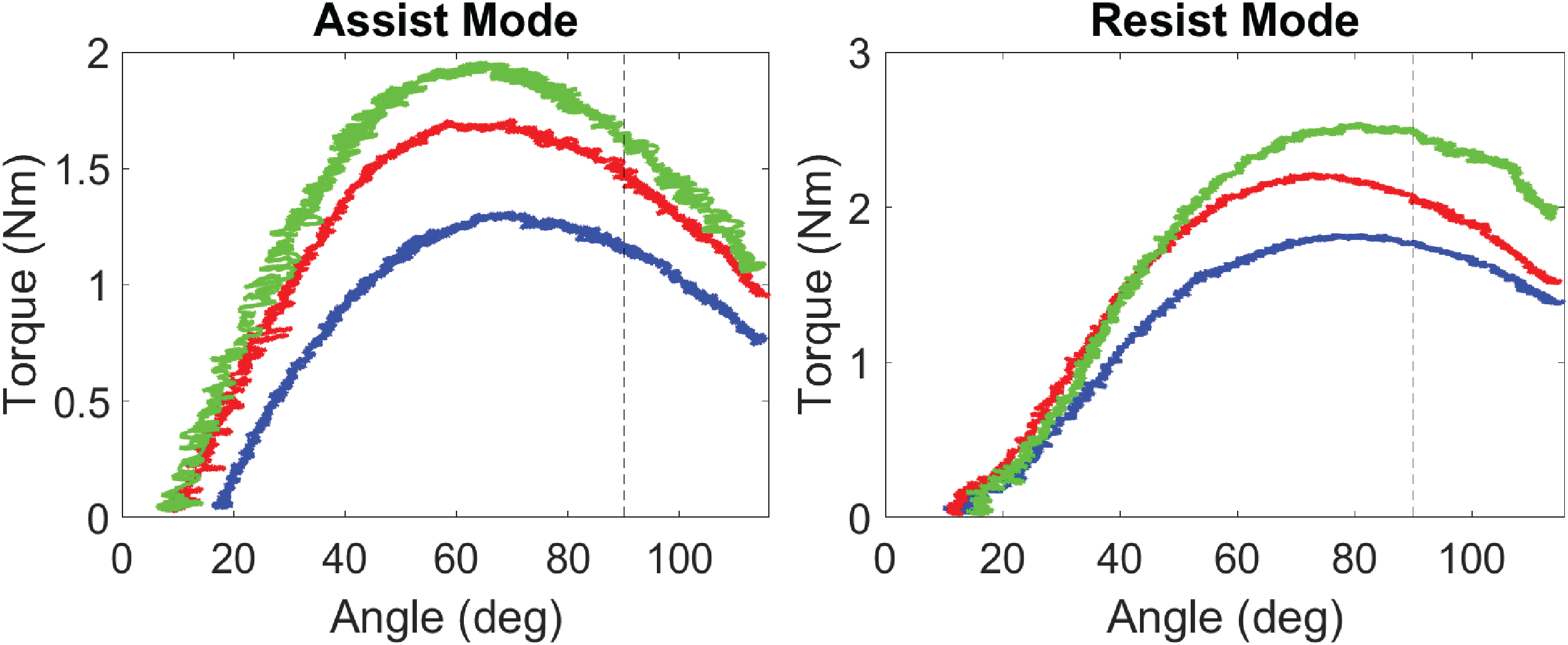


Figure S3: Torque vs. Angle profiles for the exoskeleton device in assist mode (left panel) and resist mode (right panel). Zero degrees corresponds to the arm piece at neutral (anatomical position) and 90 degrees, indicated by the dashed vertical line, corresponds to forward flexion of the arm piece to parallel to the ground. The different colors represent progressively more rubber bands.

We repeated testing using all combinations of rubber band configurations ranging from 1-3 x 7”x5/8”, 1-2 x 5”x5/8” and 1-5 x 7”x1/8”. We then calculated the peak torque for each rubber band combination and plotted them against the number of rubber bands. An example graph of the resist mode using 1 x 7”x5/8” and 1-5 x 7”x1/8” rubber bands and the assist mode using 2 x 5”x5/8” and 1-5 x 7”x1/8” rubber bands is shown in Figure S4.

The final calculations yielded the following linear relationships

$$\tau_{assist}=1.089N_{5"x5/8"}+0.16N_{7"x1/8"}-0.13 (Nm)$$

and

$$\tau_{resist}=0.722N_{7"x5/8"}+0.17N_{7"x1/8"}+0.7 (Nm)$$

where N is the number of rubber bands with subscript indicating length and width (i.e., $N_{5"x5/8"}$ is the number of 5”x5/8” rubber bands). For example, the rubber band configuration for one participant, a 77-year-old female with arm length of ~0.57m and weight ~75kg, was:

**Assist mode:** 5x5”x5/8” + 2x7”x1/8” = 5.64 Nm, which is approximately 50.7% of the torque due to gravity of her fully outstretched arm.

**Resist mode:** 6x7”x5/8” + 3x7”x1/8” = 5.54 Nm, which is approximately 49.8% of the torque due to gravity of her fully outstretched arm.


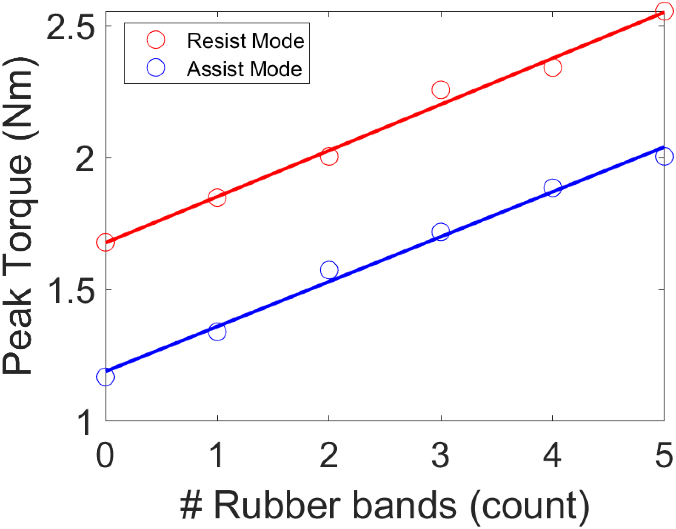


Figure S4: Peak torque as a function of the number of rubber bands in either assist mode (blue) or resist mode (red).

**Results**

*Tradeoff analysis*

Illustrated in Figure 5 (main text), RC increased by more than 1% in 6 participants and decreased by more than 1% in another 3. The absolute change in RC was less than 1% in the remaining 5 participants. Please note that ΔRC values are identical for the two muscle subplots. Deltoid MC decreased from Pre to Loading blocks by more than 3% in 9/14 of participants; deltoid MC increased by more than 3% in 2 participants. The absolute change in deltoid MC was less than 3% in the remaining 3 participants. Bicep MC decreased by more than 3% in 6 participants; bicep MC increased by more than 3% in 5 participants. The absolute change in bicep MC was less than 3% in the remaining 3 participants.

**References**

1. Brunfeldt AT, Dromerick AW, Bregman BS, Lum PS. A tradeoff between kinematic and dynamic control of bimanual reaching in virtual reality. J Neurophysiol. 2022 Apr 7;

2. McGinnis P. Biomechanics of Sport and Exercise. 3rd ed. Champaing, IL: Human Kinetics; 2013.
